# Investigation of the transcriptomic and metabolic changes associated with superficial scald physiology impaired by lovastatin and 1-methylcyclopropene in pear fruit (cv. “Blanquilla”)

**DOI:** 10.1038/s41438-020-0272-x

**Published:** 2020-04-01

**Authors:** Jordi Giné-Bordonaba, Nicola Busatto, Christian Larrigaudière, Violeta Lindo-García, Gemma Echeverria, Urska Vrhovsek, Brian Farneti, Franco Biasioli, Concetta De Quattro, Marzia Rossato, Massimo Delledonne, Fabrizio Costa

**Affiliations:** 1 XaRTA-Postharvest, Institute for Food and Agricultural Research and Technology (IRTA), Edifici Fruitcentre, Parc Científic i Tecnològic Agroalimentari de Lleida, 25003 Lleida, Spain; 2 Department of Genomics and Biology of Fruit Crops, Research and Innovation Centre, Fondazione Edmund Mach, via Mach 1, 38010 San Michele all’Adige, Trento, Italy; 3 Department of Food Quality and Nutrition, Research and Innovation Centre, Fondazione Edmund Mach, via Mach 1, 38010 San Michele all’Adige, Trento Italy; 4 Department of Biotechnology, University of Verona, Strada le Grazie 15, 37134 Verona, Italy; 5 Center Agriculture Food Environment, University of Trento, via Mach 1, 38010 San Michele all’Adige, Trento Italy

**Keywords:** Plant hormones, Abiotic

## Abstract

To elucidate the physiology underlying the development of superficial scald in pears, susceptible “Blanquilla” fruit was treated with different compounds that either promoted (ethylene) or repressed (1-methylcyclopropene and lovastatin) the incidence of this disorder after 4 months of cold storage. Our data show that scald was negligible for the fruit treated with 1-methylcyclopropene or lovastatin, but highly manifested in untreated (78% incidence) or ethylene-treated fruit (97% incidence). The comparison between the fruit metabolomic profile and transcriptome evidenced a distinct reprogramming associated with each treatment. In all treated samples, cold storage led to an activation of a cold-acclimation-resistance mechanism, including the biosynthesis of very-long-chain fatty acids, which was especially evident in 1-methylcyclopropane-treated fruit. Among the treatments applied, only 1-methylcyclopropene inhibited ethylene production, hence supporting the involvement of this hormone in the development of scald. However, a common repression effect on the *PPO* gene combined with higher sorbitol content was found for both lovastatin and 1-methylcyclopropene-treated samples, suggesting also a non-ethylene-mediated process preventing the development of this disorder. The results presented in this work represent a step forward to better understand the physiological mechanisms governing the etiology of superficial scald in pears.

## Introduction

Pears after harvest are routinely cold-stored to guarantee long-term availability satisfying the year-round market demands. Cold storage can however trigger the development of postharvest disorders mainly referred as chilling injuries, such as superficial scald^1–5^. Symptoms of superficial scald are consistent among most susceptible varieties of pome fruit, and shown as brown–dark patches on the fruit skin, resulting from the necrosis of the epidermis and hypodermal cortical tissues^1,3^. While the molecular and physiological mechanisms underlying scald in apples have been largely studied over the past few years^2,3,6–8^, scarce information is available regarding this disorder in pears. The most accepted theory for scald development focuses on the auto-oxidation of the acylic sesquiterpene α-farnesene into conjugate trienols (CTols), which leads to the production and release of the ketone 6-methyl-5-hepten-2-one^4,9–11^. In this scenario, the plant hormone ethylene has been considered as an important mediating factor through the control of the *α-farnesene synthase* gene (*AFS*)^1,2,10–15^. Nevertheless, the physiological basis of scald has been recently revisited, suggesting complex metabolic changes being involved in the development of the disorder. Among them, the oxidation of chlorogenic acid by the enzyme polyphenol oxidase (PPO) was one of the most important process correlated to the development of the scald symptoms^3,8^. Storage at low temperature can compromise the integrity of the cellular organelles’ internal membranes, promoting the reaction between PPO (stored in the plastid) and chlorogenic acid (stored in the vacuole), leading to the production of quinones and melanin, which finally leads to the dark-discolored areas on the fruit skin.

The application of the ethylene competitor 1-methylcyclopropene (1-MCP) is among the most effective strategies to prevent scald. The efficacy of 1-MCP was initially assigned to its role in reducing α-farnesene production by downregulating the expression of the *α-farnesene synthase 1* gene (*AFS1*). However, recent findings^3,8^ support that 1-MCP protects apples from developing scald not only by inhibiting ethylene-related physiological processes, but also by stimulating complex cold-acclimation reactions within the fruit. For instance, 1-MCP induced the expression of the *sorbitol-6-phosphate dehydrogenase*^8^, a key element in the production of the cryoprotectant compound sorbitol^16^. 1-MCP, moreover, induced a higher accumulation of unsaturated long-chain fatty acids within the fruit skin, essential compounds to stabilize the internal membranes, together with lower expression of *PPO*^8^.

An ethylene-independent regulation of scald has also been acknowledged, since treatment with diphenylamine (DPA)^7^ or storage under very low oxygen-modified atmospheres^5^ generally controls scald development in pome fruit, without inhibiting the fruit ethylene production capacity. Earlier studies^17^ also showed that treatment of apples and pears with lovastatin, an inhibitor of the 3-hydroxy-3-methylglutaryl-coenzyme A reductase (HMG-CoA reductase), responsible for the conversion of HMG-CoA to mevalonate, controlled scald without altering the fruit-ripening physiology. Consistent with these observations, a non-ethylene-dependent accumulation of α-farnesene has also been reported in 1-MCP-treated “Blanquilla” pears^3^, which together with other factors (i.e., influence of the fruit maturity at harvest on scald development)^4,18^ revealed the complex and likely differential regulation of this physiological disorder when comparing apples and pears. Specific studies conducted on pears have also suggested that changes in the expression of *glutathione S-transferases* (*GSTs*) and mainly a downregulation of *dehydroascorbate reductase* (*DHAR*) genes were linked to scald development^19^, hence highlighting the action that ascorbate may have on the progress of this physiological disorder^20^. In turn, 1-MCP treatments inhibiting the appearance of scald not only affected the expression of genes related to ethylene metabolism, but also the expression of several *GSTs* and *glutathione peroxidase* genes in “Wujiuxiang” pears^21^.

It is therefore evident that the physiology underlying superficial scald in pears has not yet been completely elucidated. Accordingly, this study aimed to investigate the transcriptome and metabolome variation in the superficial scald-susceptible “Blanquilla” pears in response to different scald-inhibiting treatments in an effort to unravel the physiological and molecular mechanisms influencing the development of the disorder.

## Results

### Superficial scald incidence and changes in α-farnesene and ethylene production

**Fig. 1:**
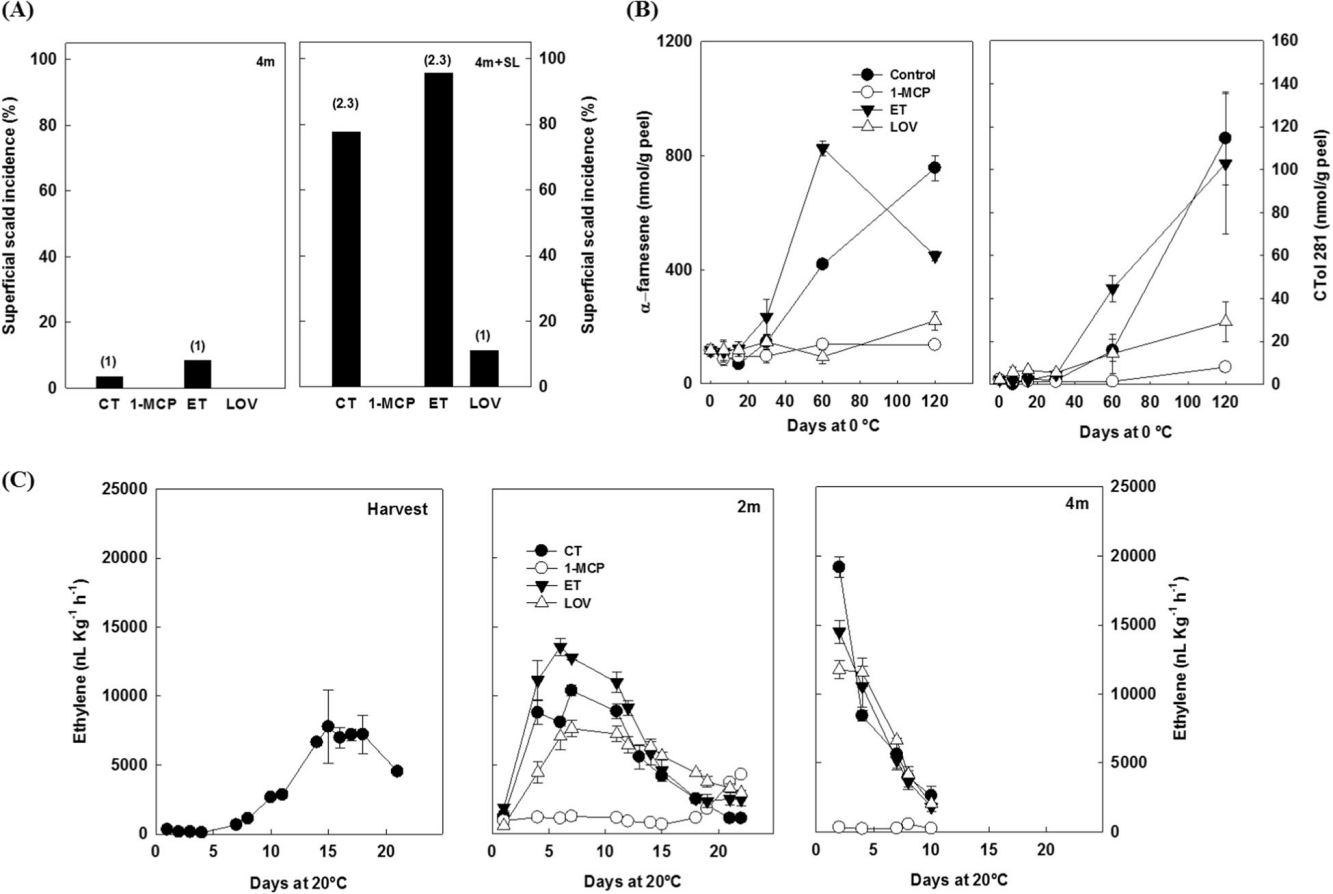
Superficial scald incidence and changes in α-farnesene and ethylene production. **a** Superficial scald incidence (% of affected fruit) and severity index (numbers within brackets above each column) in untreated “Blanquilla” pears (CT) or fruit treated at harvest, with 300 ppb of the ethylene inhibitor 1-MCP, 1.25 mmol/L of the HMGR inhibitor lovastatin, or with 200 ppm of ethylene (ET), after 4 months of cold storage, and after 4 months of cold storage plus 5 days of ripening at 20 °C (SL). **b** Changes in α-farnesene and CTol281 levels during cold storage. Error bars indicate the standard deviation (*n* = 3). (**c**) Changes in ethylene production at harvest or after 2 or 4 months of cold storage at 0.5 °C

Superficial scald was negligible for the fruit treated with 1-MCP or lovastatin upon removal from cold storage (Fig. 1). In contrast, very high incidence and severity (based on the severity scale depicted in Supplementary Fig. S1) of the disorder was detected after 5 days of shelf life both in control (CT) and ethylene-treated (ET) fruit (78%—S2.3 and 97%—S2.3, respectively, Fig. 1a; Supplementary Fig. S1). The visual inspection of the scald symptoms was in agreement with the analytical assessment of both α-farnesene and CTol281, the oxidation product of this sesquiterpene (Fig. 1b). In both cases, ethylene and 1-MCP enhanced or inhibited their accumulations, respectively. Our data showed that lovastatin impaired scald development by 85% if compared with untreated fruit (12% incidence and very low severity (S1) after 4 months + 5 days of SL), and notably reduced the accumulation of α-farnesene within the fruit skin throughout storage (Fig. 1b). The fruit ethylene production capacity upon removal from 2 or 4 months of cold storage was strongly inhibited by 1-MCP, while the rest of the treatments did not alter the ethylene production in comparison with the CT (Fig. 1c). The ethylene climacteric peak (12,000 nL kg^–1^ h^–1^) was observed for all, except for 1-MCP-treated fruit, at 7 days of shelf life following 2 months of cold storage.

### Genome-wide transcription analysis

**Fig. 2:**
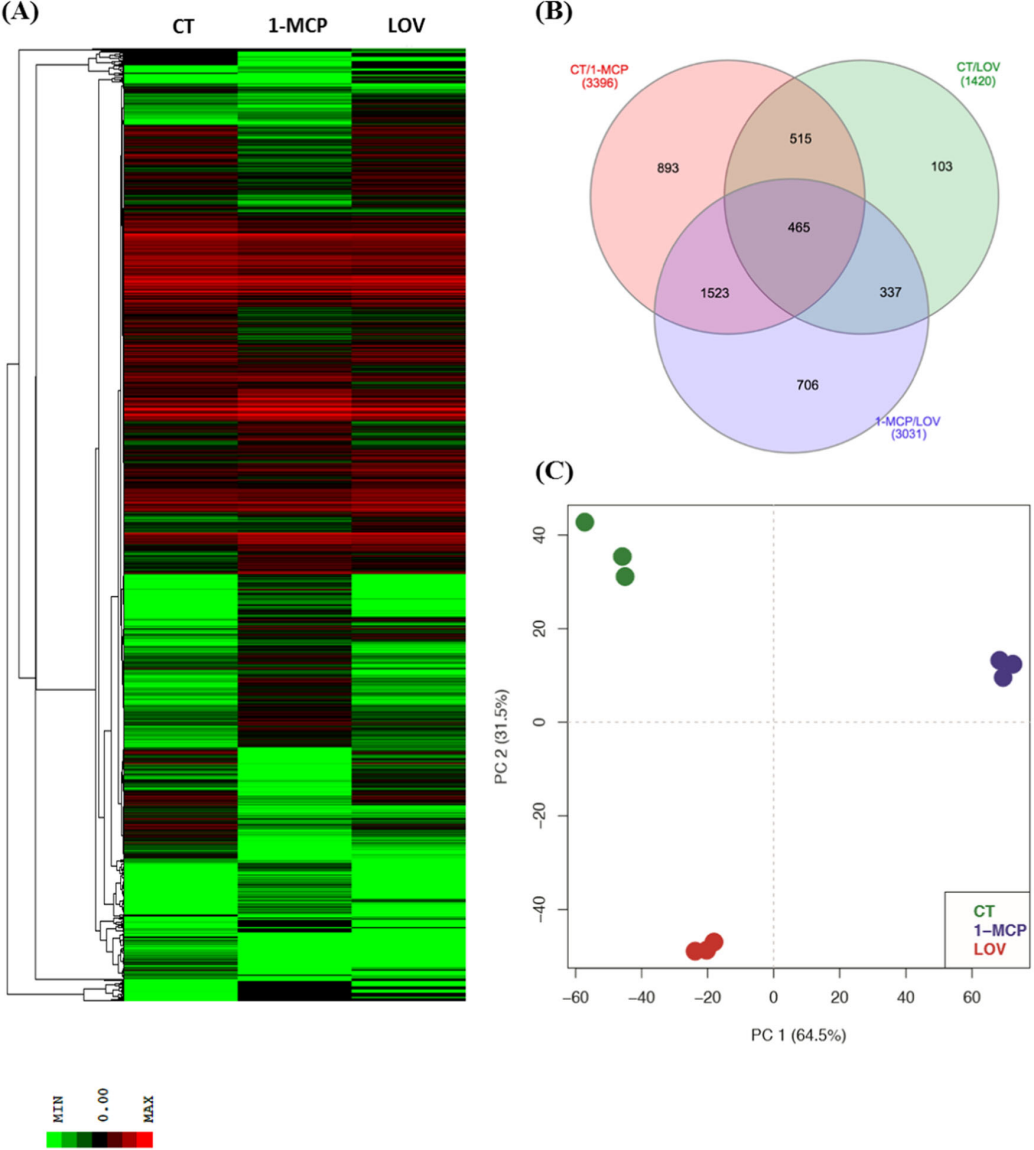
Expression profile of the DEGs across “Blanquilla” pear fruit from the different samples (CT, 1-MCP, and lovastatin) after 4 months of cold storage plus 5 days of ripening at 20 °C (SL). The expression dynamics of the DEGs is illustrated in panel (**a**) through a hierarchical clustering heatmap. **b** Venn diagram highlighting the distinctions between shared and unique genes among samples. **c** 2D-PCA plot representing the whole transcriptomic variance among the different samples. CT: control sample, 1-MCP: sample treated with 1-MCP, and lovastatin: sample treated with lovastatin

In the attempt to capture the transcriptome dynamics underlying the development of scald, and to shed light into the protecting mechanism induced by the treatments, CT together with 1-MCP and lovastatin samples was selected and used to perform a transcriptional comparison through RNA-Seq analysis. Since ET samples were phenotypically very similar to CT, in terms of scald incidence (Fig. 1a), only the latter was considered in the genome-wide transcriptome survey. To maximize the incidence of scald, fruit from the three batches was maintained at room temperature for 5 days following 4 months of cold storage. Sequencing of the RNA-Seq libraries generated a total of 418,999,237 reads (with an average of 46,555,470 reads/replicate). The alignment of the reads over the pear genome (with an average alignment rate of 93.19%) and the subsequent analysis of the differentially expressed genes (DEGs) identified 4542 uniquely assembled transcripts. From the comparison carried out across the three pairwise combinations, the application of 1-MCP led to a higher transcriptome reprogramming (Fig. 2a). The comparison between 1-MCP with CT and lovastatin samples identified 3396 (Supplementary Table S1) and 3031 DEGs (Supplementary Table 2), respectively, while the comparison made between CT and lovastatin samples identified about half of the DEGs (1420, Fig. 1b; Supplementary Table 3). The DEG expression pattern was further used to illustrate the distribution of the three samples over a 2D-PCA plot (PCA plot) defined by the first two PCs, accounting together for the 96% of the total transcriptional variance. The distribution of the samples over the plot was consistent with the number of genes detected and their expression profile. CT and lovastatin, although separated by PC2, were plotted in the negative area of PC1, while 1-MCP was projected on the opposite section of the plot, at the end of the positive PC1 quadrants (Fig. 2c).

The list of DEGs identified across the pairwise comparisons of the transcriptome profiles of the three samples highlighted groups of genes exclusively regulated under each specific condition. For instance, the downregulation of the genes involved in the ethylene biosynthesis (such as *ACS* and *ACO*) and perception and signaling (*ERS1*, *ERFs*, and *EIN3*) was unique to 1-MCP-treated samples (Supplementary Tables S1 and S2 and Supplementary Fig. S2). In contrast, in CT and lovastatin samples, the genes belonging to the ethylene domain were considerably expressed. In CT, several genes involved in the browning process typical of scald, such as the *polyphenol oxidase*, were also found to be induced. In untreated fruit, moreover, we also found the *chloroplast STAY-GREEN* gene, which was not expressed in any other of the samples investigated. In the comparison between CT and lovastatin, a much lower number of DEGs was observed, with regard to the comparison between CT and 1-MCP, suggesting that the impact of the treatment with lovastatin had a less dramatic effect on the regulation of the transcriptomic dynamics within the skin tissue. In this specific comparison, a group of genes related to the abscisic acid and gibberellin pathways (such as *gibberellin-regulated protein 1-like genes* and one *abscisic acid, 8’-hydroxylase gene*) was only upregulated in the lovastatin samples. A similar array of genes involved in the regulation of both hormones was also identified in the comparison between 1-MCP and lovastatin. In this case, each treatment was able to specifically regulate unique sets of genes belonging to the domain of both abscisic acid and gibberellins. Moreover, lovastatin was able to induce the expression of dehydrin COR47, a gene involved in the cold- acclimation process (Supplementary Table S3). In 1-MCP-treated samples, it was interesting to note the induction of genes involved in the metabolism of cutin, such as *BAHD acyltransferases*, and the transcription factor *SHINE2*, as well as genes involved in long-chain fatty acid metabolism, including *long-chain acylCoA, fatty acid hydroperoxidase lyase*, and *very-long-chain enoyl CoA reductase*. In addition, the 1-MCP treatment was able to stimulate the transcription of *temperature-induced lipocalin* and *fatty acid desaturase*, both genes being involved in the reinforcement of biological membranes under cold stress conditions, as well as in the induction of the transcription of genes related to the abscisic acid pathway and to the cutin biosynthesis.

### Expression of ethylene-related candidate genes during the modulation of the superficial scald

**Fig. 3:**
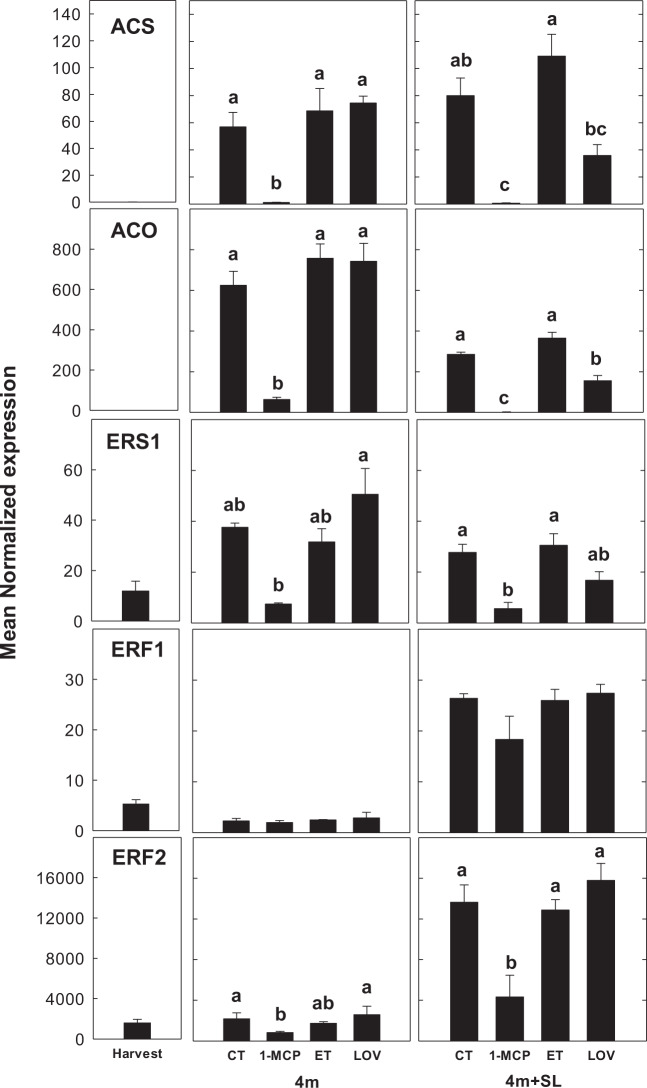
Expression profile of ethylene related genes. Rt-qPCR expression profile of candidate genes related to ethylene biosynthesis (*ACS* and *ACO*), perception (*ERS1*), and signaling transmission (*ERF1* and *ERF2*) in untreated (CT) or treated “Blanquilla” pears (1-MCP, ET, and lovastatin) after 4 months of cold storage, and after 4 months of cold storage plus 5 days of ripening at 20 °C (SL). The error bars represent the standard error of the mean- normalized expression. Different letters above each column indicate significant differences between treatments for each specific sampling (4 m or 4 m+SL). Absence of letters indicates no significant differences between treatments

The genome-wide transcription analysis identified important changes in the expression of genes involved in the ethylene-related pathway, especially in the CT/1-MCP comparison. Among the group of 18 candidate genes, selected from the DEGs according to their involvement in important pathways related to fruit ripening and scald metabolism, six were related to ethylene, including the ethylene biosynthetic (*ACS* and *ACO*) and signaling (*ERS1*, *ERS2*, *ERF1*, and *ERF2*) pathways, respectively. After 4 months of cold-storage fruit, all fruit except those treated with 1-MCP, were in a post-climacteric stage, showing a consistent decline in ethylene production (Fig. 1c). In agreement with the trend of the fruit ethylene accumulation, all six ethylene-related genes were severely downregulated in 1-MCP samples, with an induction observed after 5 days of shelf-life ripening only for *ERF1* and *ERF2* (Fig. 3). Interestingly, the application of lovastatin did not change the expression of any of the ethylene-related genes after 4 months of cold storage if compared with CT (Fig. 3), depicting a pattern in agreement to the fruit ethylene production profile (Fig. 1c). This said, notable differences between lovastatin and CT were noticed upon shelf life where the expression of *ACS* (*p* = 0.0008), *ACO* (*p* < 0.0001), and *ERS1* (*p* = 0.0033) was lower in lovastatin samples than in untreated fruit (Fig. 3).

### Molecular and metabolic changes in the production of VOCs and mevalonate pathway

**Fig. 4:**
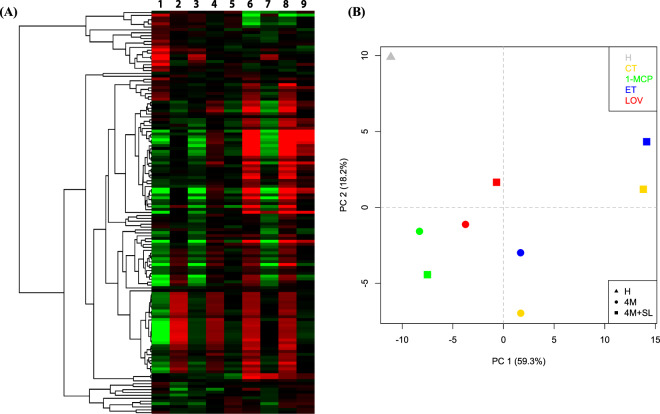
Volatile Organic Compound analysis in ‘Blanquilla’ pears. Hierarchical heatmap (panel **a**) and sample distribution over a 2D-PCA plot (panel **b**) based on the volatilome of “Blanquilla” pears at harvest, after 4 months of cold storage, or 4 months of cold storage plus 5 days of ripening at 20 °C in untreated (CT) or treated fruit (1-MCP, ethylene (ET), or lovastatin). Numbers in the hierarchical heatmap refer to 1, harvest; 2, 3, 4, and 5 for CT, 1-MCP, ET, and LOV after 4 months of cold storage, respectively; 6, 7, 8, and 9 for CT, 1-MCP, ET, and LOV after 4 months of cold storage plus 5 days of ripening at 20 °C, respectively. Each point in the PCA plot corresponds to the mean value for each group

The volatilome of the control and treated samples of “Blanquilla” pears was assessed by means of PTR–ToF–MS that identified a total of 139 volatile masses. The overall VOC production from each sample was initially depicted through a hierarchical clustering heatmap, which highlighted VOCs differentially produced by symptomatic and asymptomatic fruit (with or without scald symptoms) (Fig. 4a). CT or ET-treated fruit were characterized by a higher emission of VOCs, especially enhanced during the shelf-life period, while 1-MCP and lovastatin samples showed little change in response to storage at 20 °C (Fig. 4a). This distinction was validated by the PCA analysis carried out by implementing the first two principal components, which accounted together for 77.5% of the entire VOC variability (Fig. 4b). The 2D-plot distribution illustrated that samples collected at harvest (H), 1-MCP and lovastatin (LOV)-treated samples were plotted in the PC1-negative part of the plot, while ET-treated and CT were instead plotted on the PC1- positive part of the plot. The CT and ET-treated samples showed, moreover, a higher change in the emission of the volatilome over the ripening at 20 °C (shelf ife) if compared with samples treated with 1-MCP or lovastatin (Fig. 4b).

**Fig. 5:**
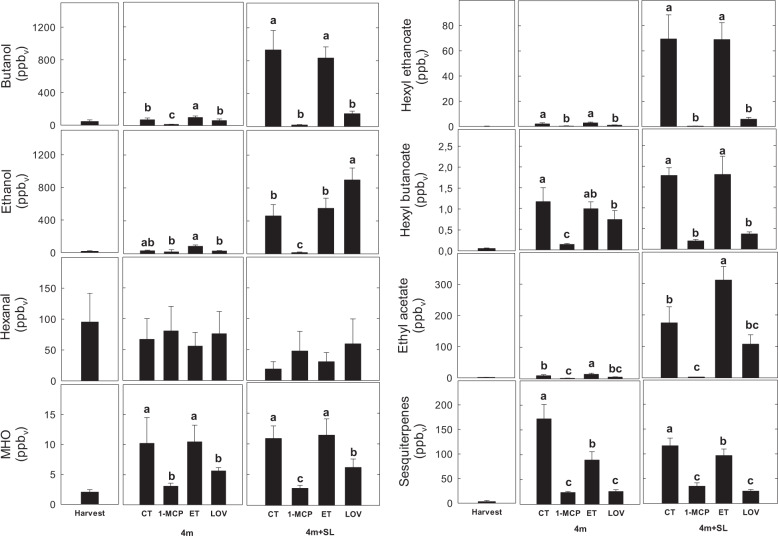
VOC concentration in ‘Blanquilla’ pears. Concentration in ppbv of eight specific volatiles, including alcohols (ethanol and butanol), aldehydes (hexanal), 6-methyl-5-hepten-2-one (MHO), esters (hexyl ethanoate, butyl hexanoate, and ethyl acetate), and sesquiterpenes in untreated “Blanquilla” pears at harvest and untreated (CT) or treated fruit (1-MCP, ethylene (ET), or lovastatin) after 4 months of cold storage, and after 4 months of cold storage plus 5 days of ripening at 20 °C (SL). Values represent the average ± standard deviation (*n* = 3). Different letters above each column indicate significant differences between treatments for each specific sampling (4 m or 4 m+SL). Absence of letters indicates no significant differences between treatments

**Fig. 6:**
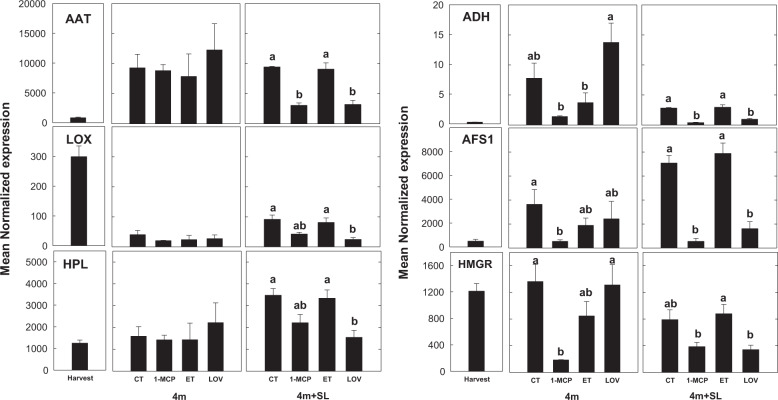
Expression of genes involved in VOC biosynthesis. Expression profile of six genes related to volatile biosynthesis (*AAT*, *LOX*, *HPL*, *ADH*, and *AFS1*) or the mevalonate pathway (*HMGR*) in untreated “Blanquilla” pears at harvest, and untreated (CT) or treated fruit (1-MCP, ethylene (ET), or lovastatin) after 4 months of cold storage, and after 4 months of cold storage plus 5 days of ripening at 20 °C (SL). The error bars represent the standard error of the mean- normalized expression. Different letters above each column indicate significant differences between treatments for each specific sampling (4 m or 4 m+SL). Absence of letters indicates no significant differences between treatments

The most important volatiles produced by “Blanquilla” fruit during ripening at 20 °C were esters and alcohols, which were the dominant VOCs in CT- and ET-treated samples. More specifically, esters, such as hexyl ethanoate, hexyl butanoate, *cis*-3-hexyl acetate, and ethyl acetate, increased as the fruit ripened (shelf life at 20 °C), and were strongly inhibited by both 1-MCP and lovastatin (Fig. 5). The observed lower amount of esters in LOV-treated fruit, similarly to 1-MCP, was related to some extent to the repression of *AAT*, *LOX*, and *HPL* genes occurring during the shelf-life period following cold storage (Fig. 6).

Ethanol was among the volatile compounds that accumulated at higher levels in lovastatin-treated fruit during shelf life, if compared with the other treatments (Fig. 5). This pattern could be related to the overexpression of the *ADH* gene (twofold higher than in untreated fruit, *p* = 0.014) occurring in the same lovastatin-treated samples in response to cold storage (Fig. 6).

### Secondary metabolite profiling

**Fig. 7:**
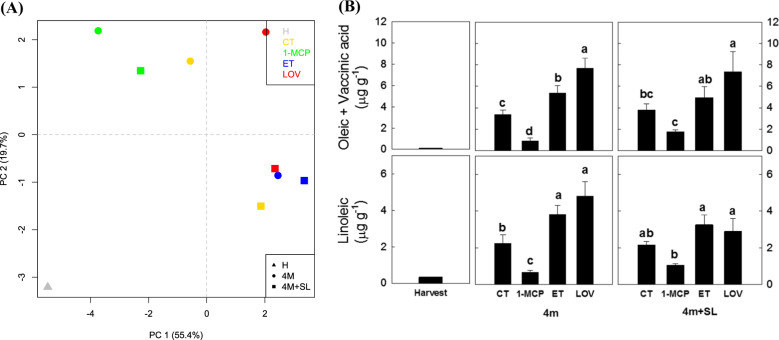
Lipid analysis in ‘Blanquilla’ pears. **a** 2D-PCA plot representing the whole variance among the different treatments based on their lipid profile. Each dot in the PCA plot corresponds to the mean value for each group. **b** Content of oleic + vaccenic acid, and linoleic acid in µg g^−1^ in untreated “Blanquilla” pears at harvest, and untreated (CT) or treated fruit (1-MCP, ethylene (ET), or lovastatin) after 4 months of cold storage, and after 4 months of cold storage plus 5 days of ripening at 20 °C (SL). Values represent the average ± standard deviation (*n* = 3). Different letters above each column indicate significant differences between treatments for each specific sampling (4 m or 4 m+SL). Absence of letters indicates no significant differences between treatments

The reprogramming of secondary metabolites, especially lipids and phenolic compounds in response to the different treatments, was also investigated (Supplementary Tables S4 and S5). For the lipid assessment, 18 compounds (Supplementary Table S4) were analyzed across the samples included in the experimental scheme. The overall variability of the phenolic compounds enabled the distinction of the pear samples over a 2D-PCA plot (Fig. 7a), taking into account the first two principal components, and explaining together 75.1% of the entire variance. The PCA plot highlighted a distinct positioning of the samples collected at the two stages (4 m and 4 m+SL) for CT and lovastatin, with the samples collected after 4 months of cold storage plotted in the PC2-negative part of the plot, and the 4 months plus 5 days at shelf life in the positive PC2 part, respectively. 1-MCP samples collected at 4 months + 5 days of shelf life were instead closely plotted to its equivalent fruit sampled at 4 months of cold storage. In contrast, the ET sample at 4 months of cold storage was plotted together with the sample collected after 5 days of additional shelf life. From the analysis of the accumulation profile for each single compound, it is interesting to note the differences in the accumulation of oleic+*cis*-vaccenic acid and linoleic acid (Fig. 7b) among the different samples. If compared with untreated fruit (CT), the application of the ethylene inhibitor induced a lower accumulation of these lipids, whereas the treatment with both ethylene and lovastatin stimulated, instead, their accumulation during cold storage and further shelf life (Fig. 7b).

**Fig. 8:**
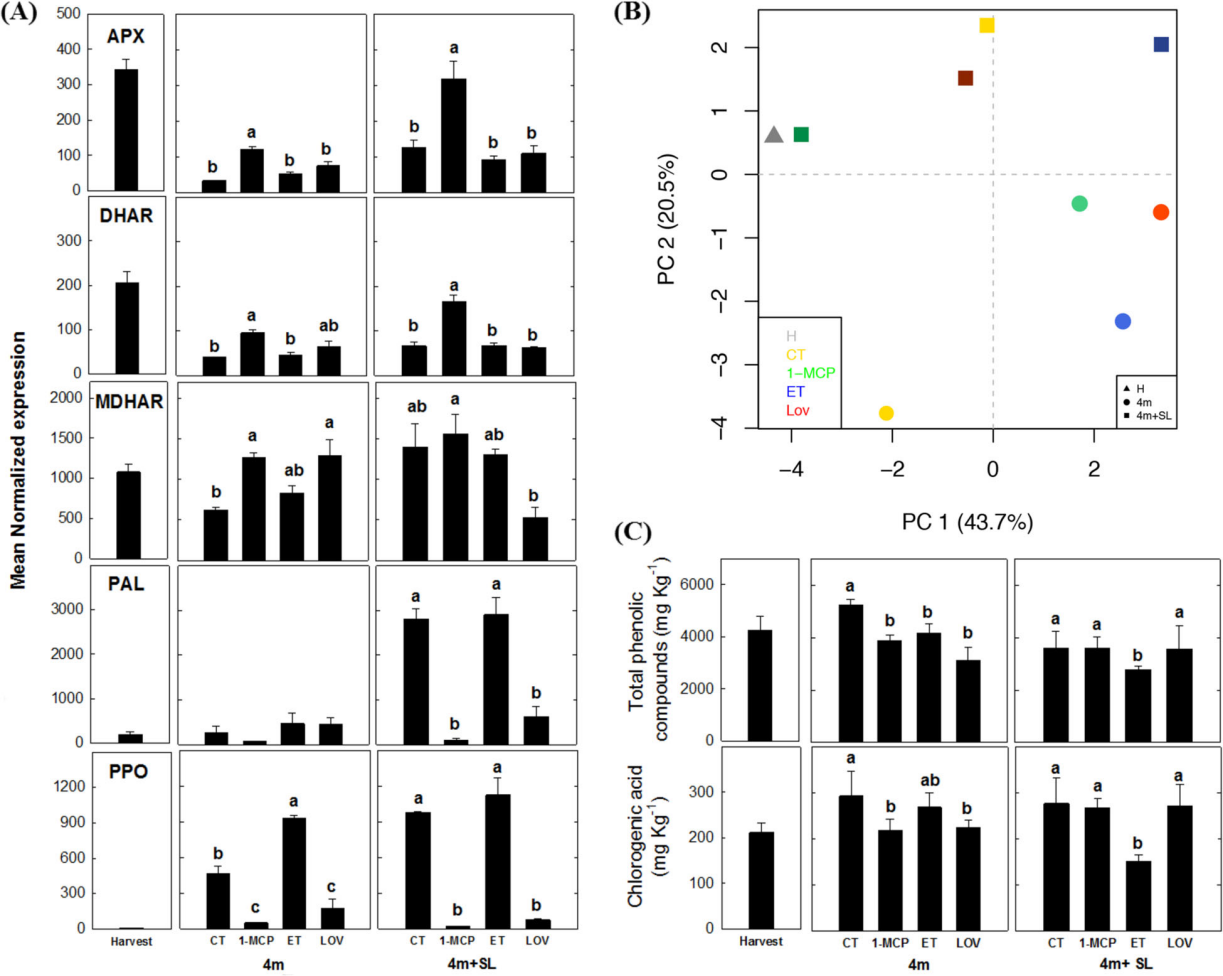
Analysis of genes and metabolites related to scald development in ‘Blanquilla’ pears. Expression profile of genes related to the ascorbate/glutathione pathway (APX, DHAR, and MDHAR), oxidation of polyphenolic compounds (PPO), or biosynthesis of polyphenolic compounds (PAL) in untreated “Blanquilla” pears at harvest, and untreated (control) or treated fruit (1-MCP, ethylene (ET), or lovastatin) after 4 months of cold storage, and after 4 months of cold storage plus 5 days of ripening at 20 °C (SL). The error bars represent the standard error of the mean-normalized expression (panel **a**). 2D-PCA plot representing the whole variance among the different treatments based on their phenolic compound profile (panel **b**). Content of total phenolic compounds and chlorogenic acid in untreated “Blanquilla” pears at harvest, and untreated (CT) or treated fruit (1-MCP, ethylene (ET), or lovastatin) after 4 months of cold storage, and after 4 months of cold storage plus 5 days of ripening at 20 °C (SL) (panel **c**). Values represent the average ± standard deviation (*n* = 3). Different letters above each column in panels **a** and **c** indicate significant differences between treatments for each specific sampling (4 m or 4 m+SL). Absence of letters indicates no significant differences between treatments

In addition to lipids, the change in the accumulation of phenolic compounds (Supplementary Table S5), and the expression of genes involved in antioxidant-related metabolism, was also investigated. Indeed, the set of DEGs targeted through the genome-wide transcriptome analysis highlighted the importance of genes involved in the ascorbate/glutathione pathway, as well as those involved in the synthesis (*PAL*) or oxidation (*PPO*) of phenylpropanoids (Fig. 8a). The three genes involved in the ascorbic acid metabolism assessed herein (*ascorbate peroxidase APX, dehydroascorbate reductase DHAR*, and *monodehydroascorbate reductase MDHAR*) resulted differentially regulated in all the samples. In lovastatin and 1-MCP samples, the three genes were overexpressed during storage, whereas a sharp upregulation of both APX and DHAR genes was found exclusively for the 1-MCP samples during shelf life (Fig. 8a). In contrast, *PAL* and *PPO* were strongly inhibited by both 1-MCP and lovastatin, a result especially evident after shelf life. The overall phenolic accumulation was depicted over a 2D-PCA plot (PCs: 64.2%), which showed a clear separation of the samples according to the sampling time (cold storage and shelf life) around the PC2 (Fig. 8b). The quantification of the total phenolics, as well as the specific assessment of the chlorogenic acid (the main substrate of PPO), especially after the cold storage, showed a pattern consistent with the expression of *PAL* and *PPO*. This compound showed a lower accumulation in the two samples not developing scald (1-MCP and lovastatin, respectively) (Fig. 8c).

### Sorbitol accumulation and biosynthesis

**Fig. 9:**
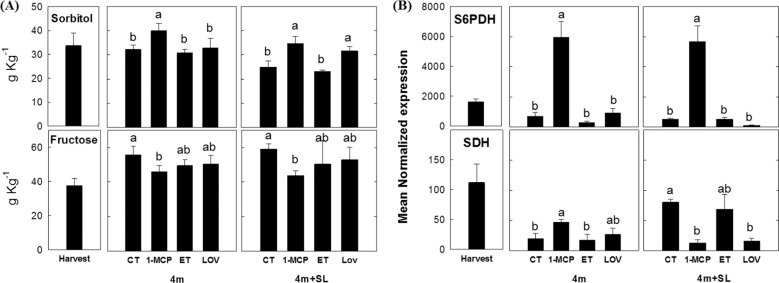
Content of sorbitol and fructose (g kg^–1^) and related gene expression. Values represent the average ± standard deviation (*n* = 3) (panel **a**) and expression profile of genes related to the biosynthesis (*S6PDH*) or degradation (*SDH*) of sorbitol in “untreated “Blanquilla” pears at harvest, and untreated (CT) or treated fruit (1-MCP, ethylene (ET), or lovastatin) after 4 months of cold storage, and after 4 months of cold storage plus 5 days of ripening at 20 °C (SL). The error bars represent the standard error of mean-normalized expression (panel **b**). Different letters above each column indicate significant differences between treatments for each specific sampling (4 m or 4 m+SL). Absence of letters indicates no significant differences between treatments

The sample treated with 1-MCP showed the highest accumulation of sorbitol (both upon removal from cold storage and after cold storage + SL) and the lowest concentration of fructose (Fig. 9a). The higher accumulation of sorbitol in 1-MCP-treated fruit was further supported by the higher transcription of *S6PDH* gene (Fig. 9b), which is known to reduce glucose into sorbitol, together with a downregulation of the *SDH* gene, encoding the enzyme responsible to convert sorbitol into fructose. Higher sorbitol concentration, if compared with untreated fruit, was also observed in lovastatin-treated samples (1.5-fold higher) upon removal from cold storage and shelf life, although not being associated with a de novo synthesis (*S6PDH*), but rather to a lower conversion of this polyol into fructose via the downregulation of the *SDH* gene (Fig. 9b).

## Discussion

### Both 1-methylcyclopropene and lovastatin inhibited superficial scald

Our results show that application of the ethylene inhibitor 1-MCP in “Blanquilla” fruit clearly limited the appearance of scald by inhibiting ethylene production and the accumulation of α-farnesene (Fig. 1). However, an ethylene-independent regulation of scald was also evident, since treatment with lovastatin, the HMG-CoA reductase inhibitor, controlled the development of this disorder without altering the fruit ethylene production (Fig. 1c). The data presented in this work further support that the production of ethylene was not impaired in fruit treated with lovastatin, which, however, showed a reduced accumulation of α-farnesene (Fig. 1b). In fact, as already reported by others^17^, lovastatin acts by interfering with the conversion of HMG-CoA to mevalonate by blocking the upper mevalonate pathway, and therefore leading to a reduced amount of substrates needed for the biosynthesis of α-farnesene^17^.

### Genome-wide transcription analysis unravels the induction of a cold-acclimation mechanism to protect pears against superficial scald

The higher transcription induction promoted by the application of 1-MCP is consistent with previous findings^8,22^, regarding the role of 1-MCP in stimulating or de-repressing specific gene expression profiles in pome fruit. The different number of DEGs observed in the comparison between CT and the two treated samples (1-MCP and lovastatin), is also in agreement with the different mode of action of these two molecules. While 1-MCP interacts with the ethylene signaling and perception system, lovastatin reduces the availability of substrates for the mevalonate pathway. The upstream interference in a fundamental hormonal signaling pathway, such as ethylene in response to 1-MCP, would likely stimulate wider downstream modifications, especially in climacteric fruit such as “Blanquilla” pear. In contrast, the variation caused by the application of lovastatin, whose action is more focused on a specific biosynthetic step, was less pronounced. The complexity of the mevalonate (MVA) pathway is, however, undebatable since this specific pathway controls the biosynthesis of hundreds of isoprenoids (including sterols and sesquiterpenes) with multiple biological functions within the plant^23^.

In the transcriptome comparison carried out in this work, two main mechanisms induced by cold storage were depicted. In CT and lovastatin-treated samples a transcriptome reprogramming was likely oriented to mainly preserve the chloroplast functionality, whereas treatment with 1-MCP stimulated instead a set of genes promoting cutine reinforcement and the stabilization of cellular membranes. In untreated fruit, general cold-acclimation responses included the expression of *STAY-GREEN*, *fatty acid desaturase, fatty acid hydroperoxidase lyase*, and *low-temperature-induced cysteine proteinase* genes. The *STAY-GREEN (SGR)* gene, in particular, is involved in the negative regulation of the chlorophyll breakdown following the chloroplast dismantling^24^, which is the first process occurring during both fruit senescence and chilling injuries. The *fatty acid hydroperoxidase lyase*, activated during stress conditions, metabolizes highly reactive hydroperoxy fatty acids generated through the lipoxygenase pathway^25^. In lovastatin-treated fruit, a *dehydrin COR47* gene was instead identified. This specific gene encodes for a protein playing a key role in adaptative responses to several abiotic stresses, including freezing temperatures, and preventing cell dehydration and collapse^26^. As already proposed by Busatto et al.^8^ for apple, the application of 1-MCP in pear induced the expression of *BAHD acyltransferase* and *SHINE2* genes, both involved in the formation of cutin^27,28^. In the reinforcement process of the cuticular wax as well as intercellular membranes, two genes involved in the metabolism of very-long-chain fatty acids (VLCFA) were identified, such as *long-chain acylCoA* and *enoylCoA reductase*^29,30^. In the 1-MCP-treated sample, a *temperature-induced Lipocalin (TIL)*, a gene encoding a protein highly accumulated in response to freezing temperatures^31^, and likely involved in the stabilization of the cellular membranes, was also observed.

It is also interesting to note that genes involved in the α-farnesene pathway were modulated in all the investigated samples (CT, 1-MCP, LOV, and ET). For instance, in lovastatin-treated fruit, a *farnesyl pyrophosphate synthase* was identified, while in 1-MCP-treated fruit, an *isopenthenyl diphosphate isomerase* was overexpressed. The prevention of scald is however primarily ensured by the downregulation of the *polyphenol oxidase* gene, as well as by the regulation of isoprenoids in the mevalonate pathway through post-translational modification^32^. In this work, the application of both lovastatin and 1-MCP prevented the development of scald not only through the repression of the *PPO* gene, but also through an induced protection of the chloroplast. In this sense, the genes involved in the metabolism of abscisic acid and gibberellin, both synthesized in the chloroplast through the mevalonate pathway, were expressed only in fruit unaffected by scald (1-MCP and lovastatin).

### Ethylene-dependent and -independent pathways are involved in the development of superficial scald

As observed in our study (Fig. 3), as well as in others^8,18,33,34^, low-temperature storage is known to upregulate the expression of genes involved in ethylene biosynthesis and perception. 1-MCP, however, may inhibit the upregulation of most of the ethylene biosynthetic genes (i.e., *ACO* and *ACS*) showing a similar response pattern than in non-chilled or just-harvested fruit^8,18,33^. In line with the ethylene production pattern (Fig. 1c), most of the ethylene-related genes measured herein were severely downregulated by 1-MCP, with a slight induction of the genes involved in ethylene perception and signaling (*ERS2, ERF1*, and *ERF2*) (Fig. 3) after 5 days of ripening at 20 °C following 4 months of cold storage. The observed induction in the expression of both *ACS* and *ACO* in response to an ethylene application is consistent with an autostimulatory climacteric process (system 2)^35^. In this scenario, it is worth noting that the application of lovastatin did not affect the regulation of these elements if compared with untreated fruit (Fig. 3), and thereby agrees with the ethylene production pattern described earlier (Fig. 1c). This result further demonstrates that the application of an HMG-CoA reductase inhibitor prevents the development of scald without affecting the fruit ethylene production, or the expression of key genes involved in ethylene metabolism.

### Molecular and metabolic changes in the production of VOCs and mevalonate pathway are associated with the superficial scald disorder in pear

The change of the volatilome in pear fruit during on- or off-tree ripening is already well documented^36–38^. In agreement with these studies, the most important volatiles produced by “Blanquilla” fruit during ripening were esters and alcohols, which resulted to be the VOCs detected at higher concentrations in both untreated and ethylene-treated fruit. Our data showed that 1-MCP strongly inhibited the expression of the *AAT* gene (Fig. 6), hence impairing the acyl transfer to alcohols to produce esters, which is consistent with available data on fruit for which ethylene was inhibited^39,40^ or suppressed^41^. The inhibition of the ester production (especially hexyl ethanoate, hexyl butanoate, and ethyl acetate^36^) in the samples treated with lovastatin was 2- to 5-fold lower than that observed in 1-MCP-treated fruit, depending on the specific compound, (Fig. 5), and was associated with inhibition of the AAT gene expression (Fig. 6).

Besides esters, other aromatic compounds (i.e., sesquiterpenes) were present at lower concentration in lovastatin-treated fruit (Fig. 5) if compared with untreated fruit. Other two important genes, involved in the mevalonate pathway and the biosynthesis of sesquiterpenes, such as *HMGR* and *AFS1*, resulted downregulated in lovastatin-treated fruit in comparison with untreated fruit, and specially during shelf life (Fig. 6). Indeed, both genes, especially after 5 days of shelf life following cold storage, were equally repressed by lovastatin and 1-MCP treatments. The application of lovastatin reduces the availability of the substrate HMG-CoA for the production of mevalonate, thus influencing the downstream conversion of farnesyl diphosphate into α-farnesene operated by the *α-farnesene synthase 1* gene (*AFS1*). The likewise downregulation of *AFS1* by 1-MCP led to corroborate the putative role of ethylene in the control of the production of this sesquiterpene^1^, although a clear connection between these two physiological pathways (mevalonate and ethylene) is not yet fully elucidated.

The higher ethanol content in lovastatin-treated fruit (1.7-fold higher than in untreated fruit) is consistent with the higher expression of the *ADH* gene, and agrees with recent studies, suggesting that higher levels of ethanol might protect the fruit from developing scald^5,42,43^. Accordingly, it is plausible to speculate that the inhibition of scald in response to lovastatin is partly mediated by a higher accumulation of ethanol that can act as a cryoprotectant or even as a weak antioxidant^5^. In this sense, the downstream inhibition of the mevalonate pathway by lovastatin may result in higher concentrations of HMG-CoA or even 3-acetyl-CoA in the fruit, favoring the metabolic pathway responsible for converting this last compound to C_16_–C_18_ fatty acids, such as oleic or linoleic acid (Fig. 7 and Supplementary Table 4). These fatty acids can be further used for the synthesis of aldehydes, and therefore for the biosynthesis of alcohols via ADH.

### The role of antioxidants in the prevention of superficial scald

The application of the chemical antioxidants diphenylamine and ethoxyquin in apples and pears, respectively, was a common practice employed to avoid the appearance of scald, preventing the oxidation of α-farnesene to MHO^20,44–48^. During the development of scald, the skin of the untreated (CT) samples was characterized by the largest polyphenol accumulation (Fig. 8c), compounds tentatively protecting the fruit against the accumulation of reactive oxygen species (ROS)^49–51^. Among the range of phenolic antioxidants, chlorogenic acid is present at relatively high concentrations in pear skin. During cold storage, the loss of membrane integrity can lead to the reaction between chlorogenic acid, initially stored in the vacuoles, with the enzyme *PPO* (stored in the chloroplast), thereby leading to the browning coloration typical of scald^3,8^. Despite the notable differences in the polyphenolic profile of apples and pears^3,52,53^, the results from this study showed that the accumulation of chlorogenic acid was higher in CT- and ET-treated fruit, immediately after 4 months of cold storage, if compared with fruit treated with 1-MCP or lovastatin (Fig. 8c). Concomitantly, the expression of *PPO* was also strongly inhibited by both 1-MCP and lovastatin treatments (Fig. 8a), reinforcing the key role of this enzyme in the development of this physiological disorder^3,8^. Although apple and pear are characterized by a different level and composition of phenolic compounds, the physiological mechanism involved in the downregulation of *PPO* seems to be conserved between the two species. Besides changes in the concentration of specific antioxidants, recent studies on pears have suggested that changes in the expression of glutathione S-transferases (*GST*) gene and mainly a downregulation of *dehydroascorbate reductase* (*DHAR*) gene were linked to scald development^19^. In agreement with this hypothesis, the three genes involved in the ascorbic acid pathway, such as *ascorbate peroxidase* (*APX*)*, dehydroascorbate reductase* (*DHAR*)*, and monodehydroascorbate reductase* (*MDHAR*), were upregulated in 1-MCP samples (at both sampling points, but especially during shelf life, Fig. 8a). This result supports the active role of the ethylene inhibitor in promoting the scavenging of ROS via an upregulation of key genes of the glutathione/ascorbate pathway, but also emphasizes, as suggested by others^20^, the protective role that ascorbate may have on the development of this physiological disorder.

### Sorbitol, a key player in the prevention of the cold-induced damage leading to superficial scald

Cold acclimation in plants causes important metabolic changes. Among those, an induced accumulation of sorbitol was already described as a mechanism inducing a higher resistance to cold in peach flower bud^16^ as well as in apple fruit^8^. Indeed, carbohydrates and sugar alcohols such as sorbitol, can act as cryoprotectants of cellular structures via osmotic adjustments^54,55^. To investigate the potential preventive role of this sugar alcohol in the development of scald in pears, the amount of sorbitol and fructose was assessed together with the expression levels of *S6PDH* and *SDH* (Fig. 9). In agreement with the profile previously observed in apple^8^, treatments leading to asymptomatic fruit showed higher concentration of sorbitol (Fig. 9). For 1-MCP-treated samples, the higher concentration of sorbitol was mainly due to a sharp overexpression of *S6PDH* together with an inhibition of *SDH*, whereas in fruit treated with lovastatin, the higher levels of sorbitol were exclusively related to the inhibition of *SDH*, showing a treatment-specific effect on the modulation of the genes related to sorbitol biosynthesis. The overall effect on the sorbitol accumulation was anyway more evident in the 1-MCP-treated samples, considering that this ethylene analog modified the expression levels of both *S6PDH* and *SDH*. It is worth noting that the accumulation pattern of sorbitol in all the samples shows more dramatic changes than those observed in the corresponding transcriptional profiles. This discrepancy was already observed in Busatto et al.^8^, and can be explained by the complex relationship occurring between the metabolite-transcript correlation as described elsewhere^56^.

## Materials and methods

### Plant materials and experimental design

“Blanquilla” pears were harvested in a commercial orchard located in the area of Lleida (North East part of Spain) at commercial maturity based on local grower standards^5^ (Firmness = ; Total soluble solids = 13.8 ± 017 °Brix, and Starch index = 6.2 ± 0.7). Immediately after harvest, fruit was sorted and selected for uniformity and absence of defects. An initial set of 30 fruits were used for initial fruit-quality evaluations (Supplementary Table S6), including fruit firmness (57.8 ± 4.7 N), starch index (6.2 ± 0.7), total soluble solids (13.8 ± 017 °Brix), and acidity (3.29 ± 0.27), measured as described elsewhere^57^. From the same fruit used for quality measurements (3 reps of a pool from 10 fruits each), the peel was removed, immediately snap-frozen with liquid nitrogen, grinded to a fine powder and stored at –80 °C prior to being used for biochemical and transcriptomic analysis. The remaining fruit was then divided into four homogeneous batches of 200 fruits each, and treated as follows prior to being stored in open crates for 4 months at 0.5 °C and 95% RH. The first batch was represented by untreated fruit and used as control (CT). The other three batches were instead treated with (i) ethylene (ET) (200 µL L^–1^) for 24 h, (ii) 1-MCP (300 nL L^–1^) applied as Smartfresh™ (Agrofresh Inc., PA, USA) for 24 h and following the company recommendations^33^, and (iii) lovastatin (1.25 mmol L^–1^, dipping for 2 min) applied as described elsewhere^17^.

As described for the samples taken at harvest, 30 fruits per treatment (3 reps of 10 fruits each) were used for standard quality measurements, as well as the peel removed for biochemical and transcriptomic analysis after 4 months of cold storage, and after 5 subsequent days of shelf-life storage (20 °C, 70–75% RH). Ninety fruits per treatment were monitored for scald after 4 months of cold storage and further 5 days of shelf life. A batch of 60 fruits per treatment (3 replicates of 5 fruits each × 5 sampling points) were used to quantify α-farnesene and CTols during storage. The remaining fruit from each treatment was employed to monitor the fruit ethylene production capacity upon removal after 2 and 4 months of storage as described below. The total number of fruit, including replicates and fruit used for each measurement and sampling, is depicted in Supplementary Fig. 3.

### Superficial scald determination and severity

Superficial scald incidence was assessed after 4 months of storage. The number of symptomatic fruit (incidence, % fruit with scald symptoms) as well as the scald severity (scale based on the % of the fruit surface showing scald symptoms; Supplementary Fig. 1) were evaluated after storage, as well as after 5 days of shelf life at 20 °C.

### Ethylene measurement

Per each treatment at harvest, and after 60 or 120 days of cold storage, the ethylene production (μL kg^−1^ h^−1^) was measured in a chamber with the temperature set at 20 °C. Two pears (per replicate and 4 replicates per treatment) were placed in a 1.5-L jar continuously ventilated with humidified air at a flow rate of 1.5 L h^−1^. One milliliter of air from the jar was taken with a syringe, and immediately injected into a GC-gas chromatograph (Agilent Technologies 6890, Wilmington, Germany) equipped with a FID detector and an alumina column 80/100 (2 m × 3 mm, Tecknokroma, Barcelona, Spain) as previously described^58^. Since “Blanquilla” pear may be in a post-climacteric ethylene phase after from 4 months of cold storage^5^, an intermediate measurement after 2 months of storage was carried out to assess any treatment effect on the production of the hormone ethylene in the full-climacteric stage.

### Spectrophotometric determination of α-farnesene and CTols

α-Farnesene and CTols were extracted, and their content analyzed following the procedure described elsewhere^59^ with slight modifications. Pear peel was isolated from the equatorial axis of the fruit (3 replicates per treatment/storage/stage and 5 fruits per replicate), and a 10-mm-diameter disk was obtained. Two excised disks/fruit were incubated in 5 mL of hexane (Panreac, Barcelona, Spain) for 10 min at 20 °C. After incubation, the hexane solution was filtered through cellulose filter paper and adjusted to 5 mL with pure hexane. After filtration, the absorbance at 232, 258, 281, and 290 nm was measured with a Uvikon 922 spectrophotometer (Kontron Instruments, Italy). The content of α-farnesene and CTol 281 was calculated as described in previous studies^15,60^, and referred to nmol g^−1^ on a fresh weight basis.

### Volatile organic compound (VOC) profiling

Determination of VOC content in pear skin tissue was performed with a PTR–ToF–MS (Ionicon Analytik GmbH, Innsbruck, Austria) in three biological replicates as described by Farneti et al.^61^. The ToF acquisition amounted to 350,000 channels for a spectrum ranging up to *m*/*z* = 400. The PTR–ToF–MS data analysis included a Poisson correction^62^. The internal calibration was operated according to Cappellin et al.^63^, and absolute headspace VOC concentrations (ppbv) were calculated as detailed elsewhere^64^. Compound annotation was carried out by comparing the spectral profile with fragmentation data of reference standards^63^.

### Epidermal lipid composition characterization

The pear skin lipid profile was characterized following the protocol reported in previous studies^65^. Extracted lipids were separated and quantified through an ultra-high-performance liquid chromatography (UHPLC) Dionex 3000 (Thermo Fisher Scientific Germany) with a RP Ascentis Express column (15 cm 9 2.1 mm; 2.7 lm C18). The UHPL was coupled to an API 5500 triple-quadrupole mass spectrometer (Applied Biosystems/MDS Sciex) with an ESI source. The lipids were identified on the base of reference standards and retention time, and further quantified as μg/g of fresh weight.

### Profiling of phenolic compounds

The analysis and characterization of phenolic compounds were carried out following the protocol described elsewhere^66^, yet with a simplified methanol–water-based sample extraction^57^. For this assessment, a UPLC Waters Acquity (Milford, MA, USA) coupled to a Waters Xevo TQMS mass spectrometer (Milford, MA, USA) was employed. Each compound was analyzed with optimized MRM conditions as previously described^66^. The softwares Waters MassLynx 4.1 and TargetLynx were employed to process the obtained phenolic data previously characterized on the base of reference compounds. The results were expressed as mg/kg of fresh weight.

### Sorbitol and fructose quantification in pear skin tissue

Sorbitol and fructose were extracted following the methodology described for apple^8^. The separation and quantification of carbohydrates were achieved by using a ICS 5000 system (Dionex-Thermo Scientific, Waltham, MA, USA). The separation was carried out with a CarboPac PA200 3 9 250-mm column, preceded by a CarboPac PA200 3 9 50-mm guard column (Dionex-Thermo Scientific, Waltham, MA, USA). Chromeleon^TM^ 7.2 Chromatography Data System software (Thermo Scientific^TM^ Dionex^TM^) was used for control processing. Sorbitol and fructose were characterized based on reference standards, and expressed as g/kg of fresh weight.

### RNA isolation and RNA-Seq library preparation

From the fruit of each condition/stage, the total RNA was extracted with the Spectrum Plant total RNA kit (Sigma-Aldrich, St. Louis, MO, USA). RNA was quantified and controlled using a NanoDrop ND8000 spectrophotometer (Thermo Scientific, Waltham, MA, USA) and a 2100 Bioanalyzer (Agilent, Santa Clara, CA, USA), respectively. To capture the effect of each treatment in modulating the development of scald, the control sample together with 1-MCP and lovastatin samples, collected after 4 months of cold storage + 5 days of shelf life at 20 °C, were employed to perform a genome-wide RNA-sequencing assay. For each replicate/sample, 3 μg of RNA (isolated from three replicates per sample) were used for the preparation of RNA-Seq library using the NEB Next Ultra II kit (BioLabs Inc., New England, following the manufacturer’s instruction). The libraries obtained here were quantified using qPCR and controlled with the DNA 1000 series II chip bioanalyzer (Agilent, Santa Clara, CA, USA). The libraries were then sequenced using an Illumina NextSeq 500 (read length of 75 bp) at the Functional Genomic Lab at the University of Verona (Italy). RNA-Seq raw data are freely available at the Gene Expression Omnibus (GEO) database with the following accession number GSE136374.

### Candidate gene RT-qPCR

Two micrograms of RNA isolated from each sample (CT, ET, 1-MCP, and LOV, collected after 4 months of cold storage, and after 4 months of cold storage + 5 days of shelf life at 20 °C) were transcribed with the SuperScript VILO cDNA Synthesis kit (Invitrogen). RT-qPCR was carried out with the ViiA7 PCR System (ThermoFisher Scientific) according to the thermal profile described elsewhere^36^. The 18 elements used in the candidate gene transcription profiling were retrieved from the set of DEGs detected through the RNA-Seq analysis or from available literature^8,19^. The set of candidate genes (listed and detailed in Supplementary Table S7) were involved in key pathways for fruit ripening and scald development in pears. Relative gene expression was represented as a mean of normalized expression, taking into account three independent Ct values. The mean-normalized expression value of each sample was calculated using the method described in previous studies^67^.

### Data analysis

RNA-Seq reads were analyzed with the cyber infrastructure CyVerse^68^. The quality of sequences for each sample was controlled with FastQC, while Scythe and Sickle were used to trim adapters and low-quality reads, respectively. The genome-wide RNA-Seq data were analyzed by implementing the New Tuxedo protocol. Reads were aligned on the pear reference genome^69,70^ through HISAT2, while StringTie was employed to assemble and quantitate RNA-Seq reads. The DEGs were detected through the R package DESeq2 with default parameters.

The expression profiles of 13 genes obtained by means of RT-qPCR were employed for validating the RNA-Seq data, showing a Pearson correlation coefficient that ranges from a minimum value of 0.755 to a maximum of 0.996.

All data regarding gene expression or metabolites were subjected to analysis of variance (ANOVA) using JMP^®^ 13.1.0 (SAS Institute Inc.). Mean comparison among treatments at each sampling stage (after cold storage or after cold storage + shelf life) was evaluated using the HSD test at a significance level of *p* < 0.05. Principal component analysis was carried out using the R package ChemometricsWithR^71^. The heatmaps were computed using the software Cluster and visualized by means of the software Java TreeView.

## Supplementary information

Figure_S1

Figure_S2

Figure_S3

Table_S1

Table_s2

Table_S3

Table_S4

Table_S5

Table_S6

Table_S7
