# Supplementary material for: Investigation of the transcriptomic and metabolic changes associated with superficial scald physiology impaired by lovastatin and 1-methylcyclopropene in pear fruit (cv. “Blanquilla”)
Source: Hortic Res. 2020 Apr 1;7:49. doi: 10.1038/s41438-020-0272-x (PMC7109095; doi:10.1038/s41438-020-0272-x)
Supplement: Supplementary file 1 — Figure_S1 [file 41438_2020_272_MOESM1_ESM.pdf]

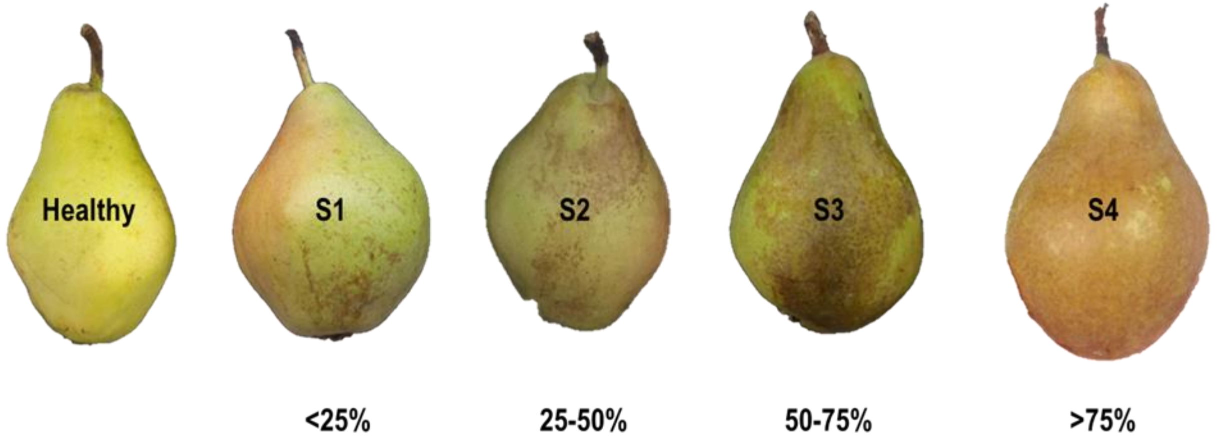

Figure S1: Scale used for the evaluation of superficial scald severity in ‘Blanquilla’ pears. The severity index (S1-S4) is based on the percentage of the fruit surface affected by the disorder.
