# Supplementary material for: Investigation of the transcriptomic and metabolic changes associated with superficial scald physiology impaired by lovastatin and 1-methylcyclopropene in pear fruit (cv. “Blanquilla”)
Source: Hortic Res. 2020 Apr 1;7:49. doi: 10.1038/s41438-020-0272-x (PMC7109095; doi:10.1038/s41438-020-0272-x)
Supplement: Supplementary file 2 — Figure_S2 [file 41438_2020_272_MOESM2_ESM.pdf]

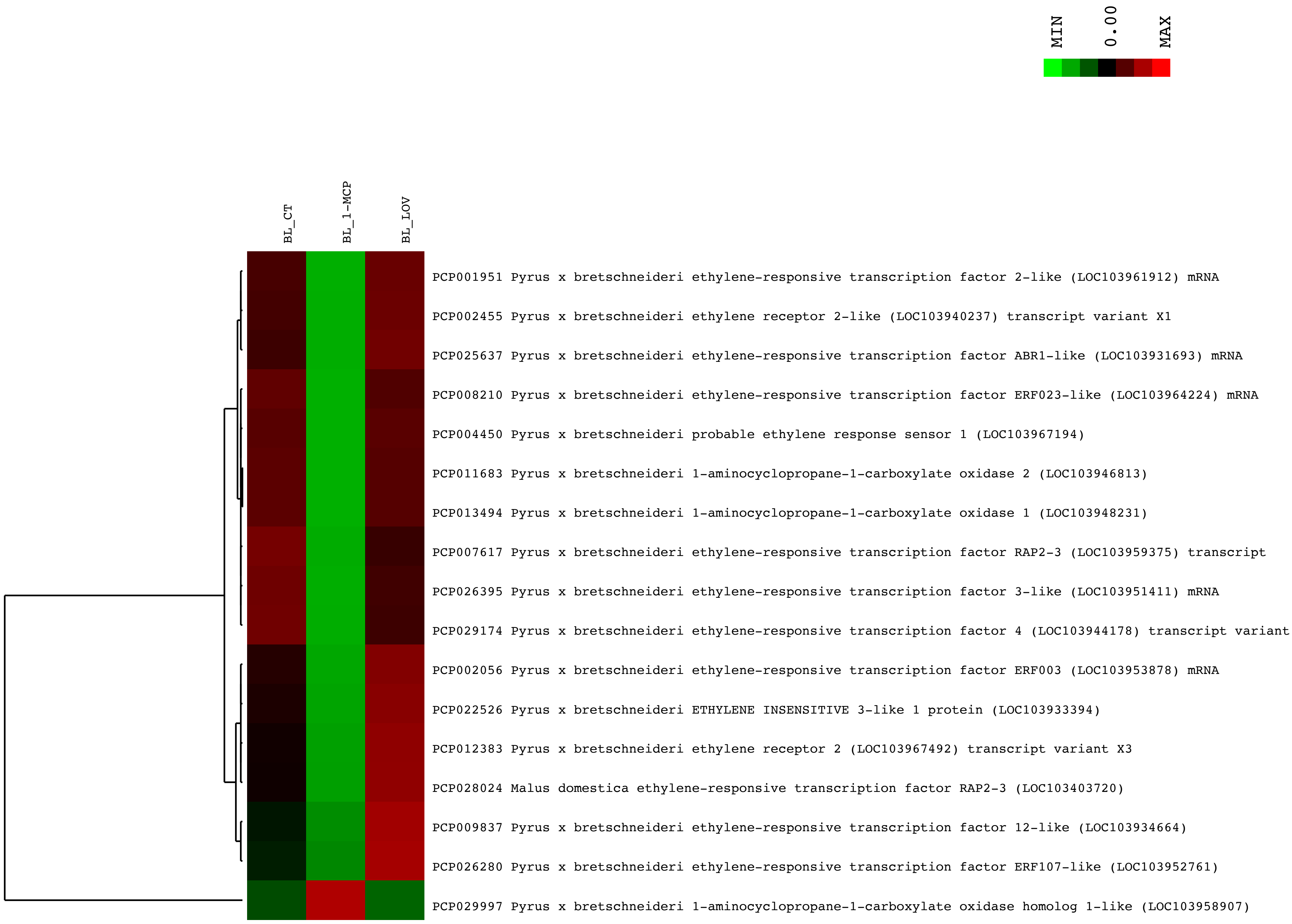

Figure S2: Hierarchical clustering heatmap representing the transcriptional dynamics of the DEGs belonging to the ethylene domain and listed both in Supplementary Table S1 and S2.
