# Supplementary material for: Investigation of the transcriptomic and metabolic changes associated with superficial scald physiology impaired by lovastatin and 1-methylcyclopropene in pear fruit (cv. “Blanquilla”)
Source: Hortic Res. 2020 Apr 1;7:49. doi: 10.1038/s41438-020-0272-x (PMC7109095; doi:10.1038/s41438-020-0272-x)
Supplement: Supplementary file 3 — Figure_S3 [file 41438_2020_272_MOESM3_ESM.pdf]

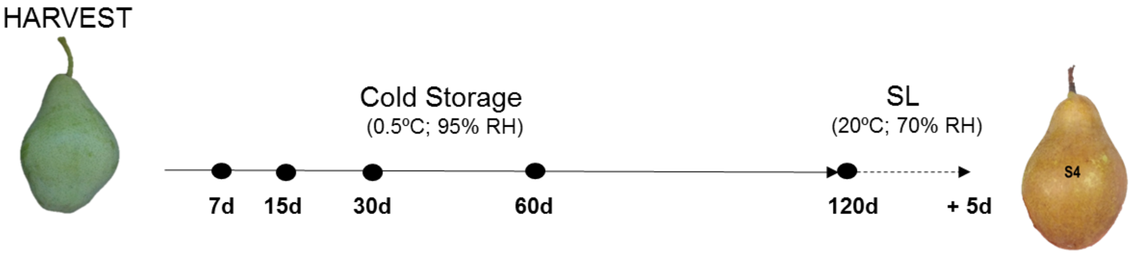

MEASUREMENT	DESCRIPTION							
Superficial scald evaluations							90*	90*
Instrumental quality		30					30	
Metabolic and molecular analysis	3 reps of 10 f (snap frozen)							
α-farnesene and CTols	3 reps of 5 f		15	15	15	15		
Ethylene measurements	4 reps of 2 f	8				8	8	
**Total nº fruit per out-turn**		38	15	15	15	23	38	90
*Same fruit used for superficial scald evaluation after 4 months of cold storage and after SL.		**196 f per treatment**						

Figure S3: Experimental design depicting the different samplings as well as the number of fruit used for each of the different measurements.
