# Supplementary material for: Investigation of the transcriptomic and metabolic changes associated with superficial scald physiology impaired by lovastatin and 1-methylcyclopropene in pear fruit (cv. “Blanquilla”)
Source: Hortic Res. 2020 Apr 1;7:49. doi: 10.1038/s41438-020-0272-x (PMC7109095; doi:10.1038/s41438-020-0272-x)
Supplement: Supplementary file 9 — Table_S6 [file 41438_2020_272_MOESM9_ESM.docx]

**Supplementary Table S6**:

|  |  | **Firmness (N)** | **SSC (%Brix)** | **TTA (g mal/l)** |
| --- | --- | --- | --- | --- |
| **H** | | 57,80 | 13,88 | 3,29 |
|  |  | (±4,73) | (±0,17) | (±0,27) |
| **4M** | **CT** | 37,09 | 13,65 | 2,21 |
|  |  | (±9,13) | (±0,21) | (±0,08) |
|  | **1MCP** | 65,14 | 13,78 | 2,50 |
|  |  | (±9,90) | (±0,26) | (±0,25) |
|  | **ET** | 26,67 | 12,88 | 2,13 |
|  |  | (±6,50) | (±0,26) | (±0,09) |
|  | **LOV** | 37,83 | 12,60 | 2,02 |
|  |  | (±7,90) | (±0,08) | (±0,22) |
| **4M + SL** | **CT** | 16,77 | 13,68 | 1,88 |
|  |  | (±2,75) | (±0,10) | (±0,16) |
|  | **1MCP** | 59,11 | 13,30 | 2,52 |
|  |  | (±5,89) | (±0,38) | (±0,35) |
|  | **ET** | 14,46 | 12,73 | 2,01 |
|  |  | (±2,64) | (±0,26) | (±0,09) |
|  | **LOV** | 18,83 | 12,50 | 2,11 |
|  |  | (±2,26) | (±0,29) | (±0,11) |
