# Supplementary material for: Investigation of the transcriptomic and metabolic changes associated with superficial scald physiology impaired by lovastatin and 1-methylcyclopropene in pear fruit (cv. “Blanquilla”)
Source: Hortic Res. 2020 Apr 1;7:49. doi: 10.1038/s41438-020-0272-x (PMC7109095; doi:10.1038/s41438-020-0272-x)
Supplement: Supplementary file 10 — Table_S7 [file 41438_2020_272_MOESM10_ESM.docx]

**Table s1**: List of primer pairs used to assess the expression pattern of 18 selected genes by RT-qPCR along with the reference gene used in the analysis

| **Gene** | **Primer name** | **Anotation** | **Sequence (5'-3')** | **Target gene** | **Metabolic pathway/Biological function** |
| --- | --- | --- | --- | --- | --- |
| ACS | Pc_ACS_for2 | Aminocyclopropane-1-carboxylic acid synthase | ATGCTGGCTTGTTCTGTTGG | PCP011500 | Ethylene biosynthesis |
|  | Pc_ACS_rev2 |  | AGGTTCCGTGCAATGACAAG |  |  |
| ACO | Pc_ACO_for1 | Aminocyclopropane-1-carboxylic acid oxidase | AAGGTCAGCAACTACCCTCC | PCP011683 |  |
|  | Pc_ACO_rev1 |  | TGTCATCCTGGAAGAGCAGG |  |  |
| ERS1 | Pc_ERS1_for2 | ethylene response sensor 1 | TGAAGTTCACAAAGCAGGGC | PCP004450 | Ethylene perception/sensing |
|  | Pc_ERS1_rev2 |  | CGTAGGTAGAAGTGGCCCTC |  |  |
| ERF1 | Pc_ERF1_for2 | ethylene response factor 1 | AACATTCGAAACGGCGGAAG | PCP015040 | Ethylene response factor |
|  | Pc_ERF1_rev2 |  | CGAGGACTGAGACGCATTTG |  |  |
| ERF2 | Pc_ERF2_for1 | ethylene response factor 2 | TGGCTTCACTCCAGATGACC | PCP002056 |  |
|  | Pc_ERF2_rev1 |  | ATATCCGGCATTTTCGCACC |  |  |
| LOX | Pc_LOX1_for1 | lipoxygenase | CTTCAACGGAGAATCAGGCG | PCP002320 | Volatiles biosynthesis |
|  | Pc_LOX1_rev1 |  | TCGGTTATGTCATCGAGGGG |  |  |
| ADH | Pc_ADH2_for1 | alcohol dehydrogenase | GTTTGTTCACCTAAGCCGGG | PCP031262 |  |
|  | Pc_ADH2_rev1 |  | TAGCAACCCGACAATTTGGC |  |  |
| AAT | Pc_AAT2_for2 | alcohol acyltransferase | AGAGGCGCAGATGCACCATC | PCP024687 |  |
|  | Pc_AAT2_rev2 |  | GTTACTGAATGCGTATGAGCCATC |  |  |
| HPL | Pc_HPL2_for2 | hydroperoxide lyase | CTCTCTCAACCACAACCACAACC | PCP012365 |  |
|  | Pc_HPL2_rev2 |  | CTCGGTGCGCTTCTTGAAG |  |  |
| AFS1 | Pc_AFS1_for | α-farnesene synthase | GAAAACTAGGCCTCGCGAAC | PCP028486 |  |
|  | Pc_AFS1_rev |  | TTCGATAGCTGCAATGCCGT |  |  |
| HMGR | Pc_HMGR_for2 | 3-hydroxy-3-methylglutaryl-coenzyme A reductase | ACGACGGCAAGGACCTTCATG | PCP017787 | Mevalonate |
|  | Pc_HMGR_rev2 |  | GCAGGCTGCTTGTGATGCAAG |  |  |
| APX | Pc_APX_for | ascorbate peroxidase | GTCCCATTCCACCCAGGAAG | PCP023254 | Glutathione/ascorbate |
|  | Pc_APX_rev |  | CCACCGGAGAGAGCAACAAT |  |  |
| DHAR | Pc_DHAR_for | dehydroascorbate reductase | TCTCGAGGTCGCTGTCAAG | PCP007080 |  |
|  | Pc_DHAR_rev |  | GGGGTTTGTCGCTGAGATT |  |  |
| MDHAR | Pc_MDHAR_for | monodehydroascorbate reductase | TGGTGTCAAAGGAGCTGATG | PCP029858 |  |
|  | Pc_MDHAR_rev |  | ACCAAGCTCGAGACCAATGT |  |  |
| PAL | Pc_PAL | phenylalanine ammonia lyase | AGACCCTCAATGCCTCAGAA | PCP027603 | Polyphenols biosynthesis/oxidation |
|  | Pc_PAL |  | CAAGCCAGAACCAACAGCAG |  |  |
| PPO | Pc_PPO_for | polyphenol oxidase | CCTACTCACAAAGCCCAAGC | PCP039035 |  |
|  | Pc_PPO_rev |  | CCTCCAAGACCAAGAAGCAC |  |  |
| S6PDH | Pc_S6PDH1_for | NADP-dependent D-sorbitol-6-phosphate dehydrogenase | CTTTGGCGTCTGGAGAAGAA | PCP013307 | Sorbitol metabolism |
|  | Pc_S6PDH1_rev |  | GTGGAGCCACATGGAGATTAG |  |  |
| SDH | Pc_SDH2_for | sorbitol dehydrogenase | ATGGTCACAGCCATTGGTCA | PCP002232 |  |
|  | Pc_SDH2_rev |  | ACCTTGTCCTTGCCCAGAAG |  |  |
| Md8283 | Md_8283_for | housekeeping | CTCGTCGTCTTGTTCCCTGA | PCP030439 | Housekeeping |
|  | Md_8283_rev |  | GCCTAAGGACAGGTGGTCTATG |  |  |
